# Synergistic effects of PA (S184N) and PB2 (E627K) mutations on the increased pathogenicity of H3N2 canine influenza virus infections in mice and dogs

**DOI:** 10.1128/jvi.01984-24

**Published:** 2025-04-04

**Authors:** Xiangyu Xiao, Xinrui Wang, Fengpei Xu, Yanting Liang, Yi Luo, Shoujun Li, Pei Zhou

**Affiliations:** 1 Guangdong Provincial Pet Engineering Technology Research Center, College of Veterinary Medicine, South China Agricultural University 554665, Guangzhou, Guangdong, People's Republic of China; Cornell University Baker Institute for Animal Health, Ithaca, New York, USA

**Keywords:** H3N2 canine influenza virus, cross-species transmission, host adaptation, single-cell RNA sequencing

## Abstract

As companion animals, dogs are susceptible to various subtypes of influenza A virus (IAV), with the H3N2 and H3N8 subtypes of canine influenza virus (CIV) stably circulating among canines. Compared to the H3N8 CIV, the H3N2 CIV is more widely prevalent in canine populations and demonstrates increased adaptability to mammals, potentially facilitating cross-species transmission. Therefore, a comprehensive elucidation of the mechanisms underlying H3N2 CIV adaptation to mammals is imperative. In this study, we serially passaged the GD14-WT strain in murine lungs, successfully establishing a lethal H3N2 CIV infection model. From this model, we isolated the lethal strain GD14-MA and identified the key lethal mutations PA(S184N) and PB2(E627K). Moreover, the GD14-ma[PA(S184N)+PB2(E627K)] strain exhibited markedly enhanced pathogenicity in dogs. Viral titers in lung tissues from infected dogs and mice showed that GD14-ma[PA(S184N)+PB2(E627K)] does not increase its pathogenicity to mice and dogs by upregulating viral titers compared to the GD14-WT strain. Notably, sequence alignments across all H3N2 IAVs showed an increasing prevalence of the PA (S184N) and PB2 (E627K) mutations from avian to human hosts. Finally, single-cell RNA sequencing of infected mouse lung tissues showed that GD14-ma[PA(S184N)+PB2(E627K)] effectively evaded host antiviral responses, inducing a robust inflammatory reaction. Considering the recognized role of the PB2 (E627K) mutation in the mammalian adaptation of IAVs, our findings underscore the importance of ongoing surveillance for the PA (S184N) mutation in H3N2 IAVs.

**IMPORTANCE:**

Since the 21st century, zoonotic viruses have frequently crossed species barriers, posing significant global public health challenges. Dogs are susceptible to various influenza A viruses (IAVs), particularly the H3N2 canine influenza virus (CIV), which has stably circulated and evolved to enhance its adaptability to mammals, including an increased affinity for the human-like SAα2,6-Gal receptor, posing a potential public health threat. Here, we simulated H3N2 CIV adaptation in mice, revealed that the synergistic PA(S184N) and PB2(E627K) mutations augment H3N2 CIV pathogenicity in dogs and mice, and elucidated the underlying mechanisms at the single-cell level. Our study provides molecular evidence for adapting the H3N2 CIV to mammals and underscores the importance of vigilant monitoring of genetic variations in H3N2 CIV.

## INTRODUCTION

Since the inception of the 21st century, zoonotic viruses have frequently surmounted species barriers, leading to numerous epidemics among human populations. The emergence of pathogens such as H1N1 influenza A virus (IAV), H5N1 and H7N9 avian influenza viruses (AIVs), Middle East respiratory syndrome coronavirus, severe acute respiratory syndrome coronavirus, Ebola virus, and the more recent severe acute respiratory syndrome coronavirus 2 have posed formidable challenges to global public health (1–7). These incidents underscore the urgent need for comprehensive research into the mechanisms of cross-species transmission and the epidemiological characteristics of these zoonotic agents.

IAV exhibits a broad host range, including humans, birds, horses, dogs, pigs, and marine mammals (8). The genome of IAV, consisting of single-stranded, segmented RNA, renders it inherently prone to genetic mutation and reassortment. This genetic plasticity is a key determinant of the virus’s enhanced virulence and its ability to transmit across species. Two principal pathways can lead to host shifts in IAV (9). The first pathway involves the direct transmission of IAVs to mammals, followed by the accumulation of adaptive mutations that result in the establishment of enduring lineages. The second pathway occurs through the co-infection of a single host with two distinct IAV strains, leading to the emergence of novel viral strains via genetic reassortment. For example, the IAV strains responsible for the human influenza pandemics in 1957, 1968, and 2009 have all undergone genetic reassortment with avian or swine IAVs (10). Pigs and birds are currently recognized as the quintessential intermediate hosts facilitating the interspecies transmission of animal IAV to humans. However, the role of dogs in the evolutionary dynamics of IAVs warrants consideration. As companion animals with close contact with humans and known susceptibility to various IAV subtypes, dogs may represent a significant, yet potentially underappreciated, interface in the ecology and epidemiology of IAV (11–18).

Dogs are susceptible to multiple subtypes of IAVs, including H5N1, H6N1, H9N2, and H10N8, thereby facilitating the isolation of diverse reassortant IAVs in this species (11, 13, 14, 17). Specifically, CIVs with the PA gene segment from H9N2 AIV, the H5N2 subtype from swine and avian influenza virus recombination, and the H3N1 subtype that underwent genetic reassortment with the pandemic H1N1 strain and the H3N2 CIV have been identified (19–21). At present, only the equine-origin H3N8 CIV and the avian-origin H3N2 CIV have achieved stable endemic circulation within canine populations (16, 18, 22). The prevalence of H3N8 CIV has significantly decreased in dogs, while the H3N2 CIV continues to circulate widely (23). Sialic acid receptors play a crucial role in IAV infection and host range determination. During adaptation to canine populations, H3N2 CIVs have evolved to recognize the human-like SAα2, 6-Gal receptor, characterized by enhanced hemagglutinin stability, increased replication in human airway epithelial cells, and a 100% transmission rate in the ferret model (24). H3N2 CIVs exhibit an expansive host tropism; experimental studies have confirmed their ability to infect species beyond dogs, including ferrets, pigs, guinea pigs, and cats, highlighting their broader zoonotic potential (25–27). Interspecies transmission of H3N2 CIV from dogs to cats has been reported in South Korea and China (28–30). No human infections with H3N2 CIV have been documented. However, experimental infections indicate that human pandemic IAV, specifically the H1N1 and H3N2 subtypes, can infect dogs, with the H3N2 subtype showing transmissibility within canine populations (31). Serological surveys also reveal antibodies against human-origin H1N1 and H3N2 IAVs in canine sera, indicating possible exposure or infection events (32–36).

In this study, we serially passaged H3N2 CIV in the lungs of BALB/c mice, resulting in a lethal strain for mice. Using reverse genetics, we confirmed the key genetic determinants of this strain and its pathogenicity in mice and dogs. In addition, single-cell sequencing analysis is also utilized to further elucidate the underlying pathogenic mechanisms.

## RESULTS

### Establishment of a lethal H3N2 CIV infection model in mice

**Fig 1:**
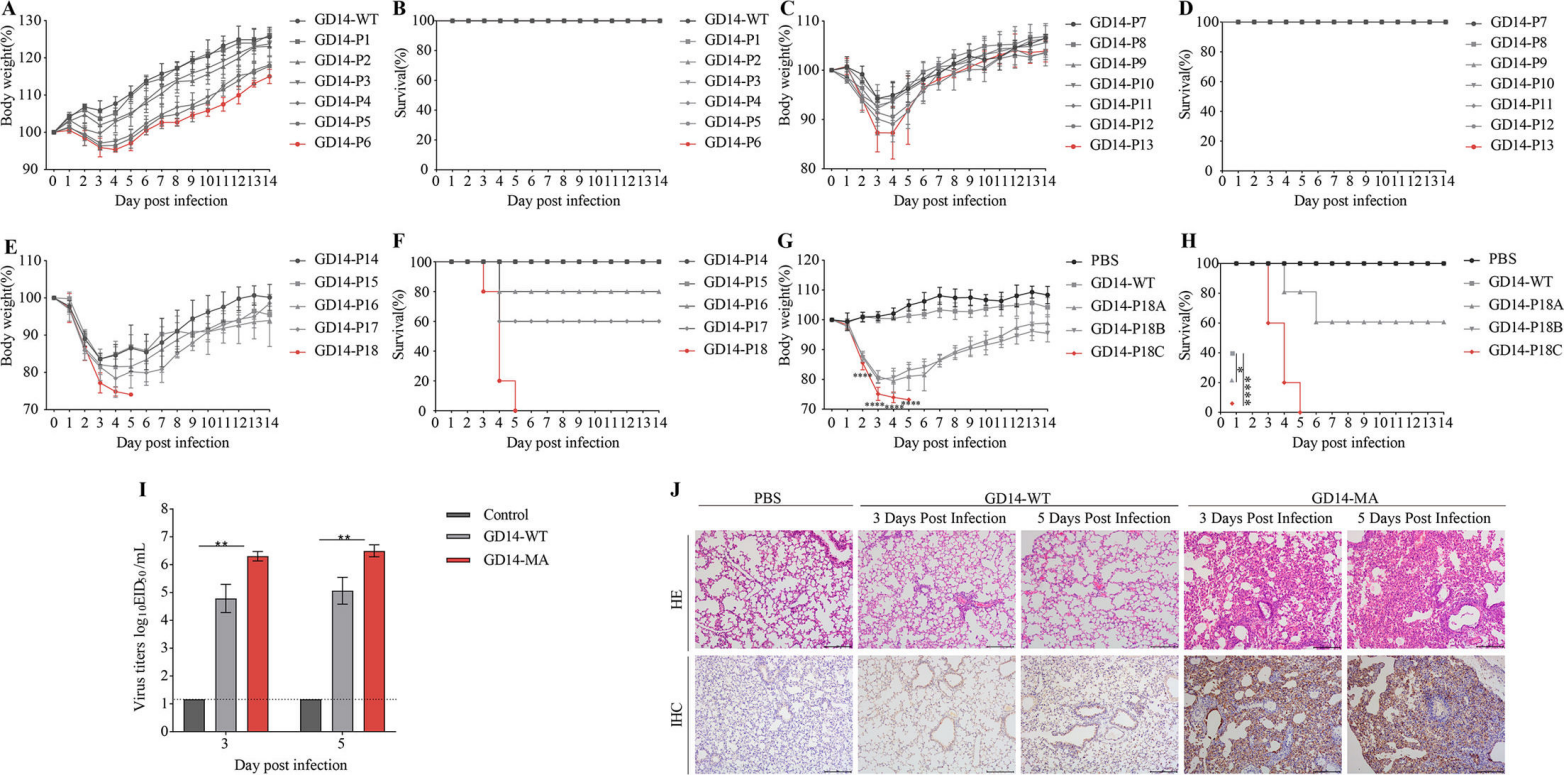
Establishment of a lethal H3N2 CIV infection model in mice. (**A and B**) Body weight changes and survival rate of 4-week-old mice. (**C and D**) Body weight changes and survival rate of 5-week-old mice. (**E and F**) Body weight changes and survival rate of 6-week-old mice. (**G and H**) Body weight changes and survival rate of mice infected with plaque-purified viral strains. (Five mice per group; mice were considered to have succumbed if they lost more than 25% of their initial body weight). (**I**) Lung viral titers at 3 and 5 dpi. The dashed line signifies the lower limit of detection. (**J**) Histological assessment of lung sections from experimental mice at 3 and 5 dpi via hematoxylin-eosin (HE) staining and immunohistochemistry (IHC).

To develop a lethal murine model of H3N2 CIV, GD14-WT was serially passaged in mice aged 4, 5, and 6 weeks, with continuous monitoring of body weight and survival rates for 14 days post-inoculation (dpi). Four-week-old mice exhibited minimal changes in body weight (Fig. 1A), whereas 5-week-old mice displayed a notable reduction in body weight by the 13th passage (Fig. 1C). The survival analysis indicated no mortality among 4-week-old (Fig. 1B) or 5-week-old mice (Fig. 1D). By contrast, 6-week-old mice showed a significant decline in body weight by the 18th passage (Fig. 1E), resulting in complete mortality by the 5th dpi (Fig. 1F).

To isolate a homogeneous lethal murine-adapted viral strain, the 18th passage of the virus was purified via plaque assay in MDCK cells. Three distinct plaque sizes, designated GD14-P18A, GD14-P18B, and GD14-P18C, were selected and propagated in specific-pathogen-free chicken embryos. GD14-P18A, GD14-P18B, GD14-P18C, and GD14-WT were then intranasally inoculated into 6-week-old mice at a viral titer of 10^6^ EID50, with a control group receiving 50 µL of PBS. Groups inoculated with GD14-P18A, GD14-P18B, and GD14-P18C (*P* < 0.0001) exhibited significantly more rapid weight loss compared to the GD14-WT group (Fig. 1G). Notably, all mice in the GD14-P18C-infected group had died by the 5th dpi (*P* < 0.0001), whereas the survival rates for the GD14-P18A (*P* < 0.05) and GD14-P18B groups were 60% and 100%, respectively (Fig. 1H). Ultimately, we sequenced the GD14-P18C strain, which exhibited higher virulence compared to GD14-P18A and GD14-P18B, and designated it as GD14-MA. To elucidate the molecular basis underlying the increased virulence of the GD14-MA strain, we performed genomic sequencing on this strain. The genomic comparison revealed eight amino acid mutations across five viral segments in GD14-MA compared to the GD14-WT (Table 1). Lung viral titers at 3 and 5 dpi were significantly higher in the GD14-MA group compared to the GD14-WT group (*P* < 0.01) (Fig. 1I). Histopathological analyses revealed that GD14-MA induced severe lobar pneumonia with fibrinous exudation and inflammatory cell infiltration, in stark contrast to the minimal changes observed in GD14-WT mice (Fig. 1J). Immunohistochemical detection of viral nucleoprotein confirmed the widespread presence of GD14-MA in infected lungs (Fig. 1J).

**TABLE 1:** Amino acid substitutions identified in GD14-MA virus protein during mouse adaptation

Protein	Position	Change of amino acids in H3N2 CIV	
		GD14-WT	GD14-MA
PB2	627	E	K
PA	100	V	I
PA	184	S	N
PA	349	E	K
HA	256	G	E
HA	486	Y	H
NA	36	Y	H
M	192	M	V

### Synergistic lethal effects of PA (S184N) and PB2 (E627K) mutations in GD14-MA murine infections

**Fig 2:**
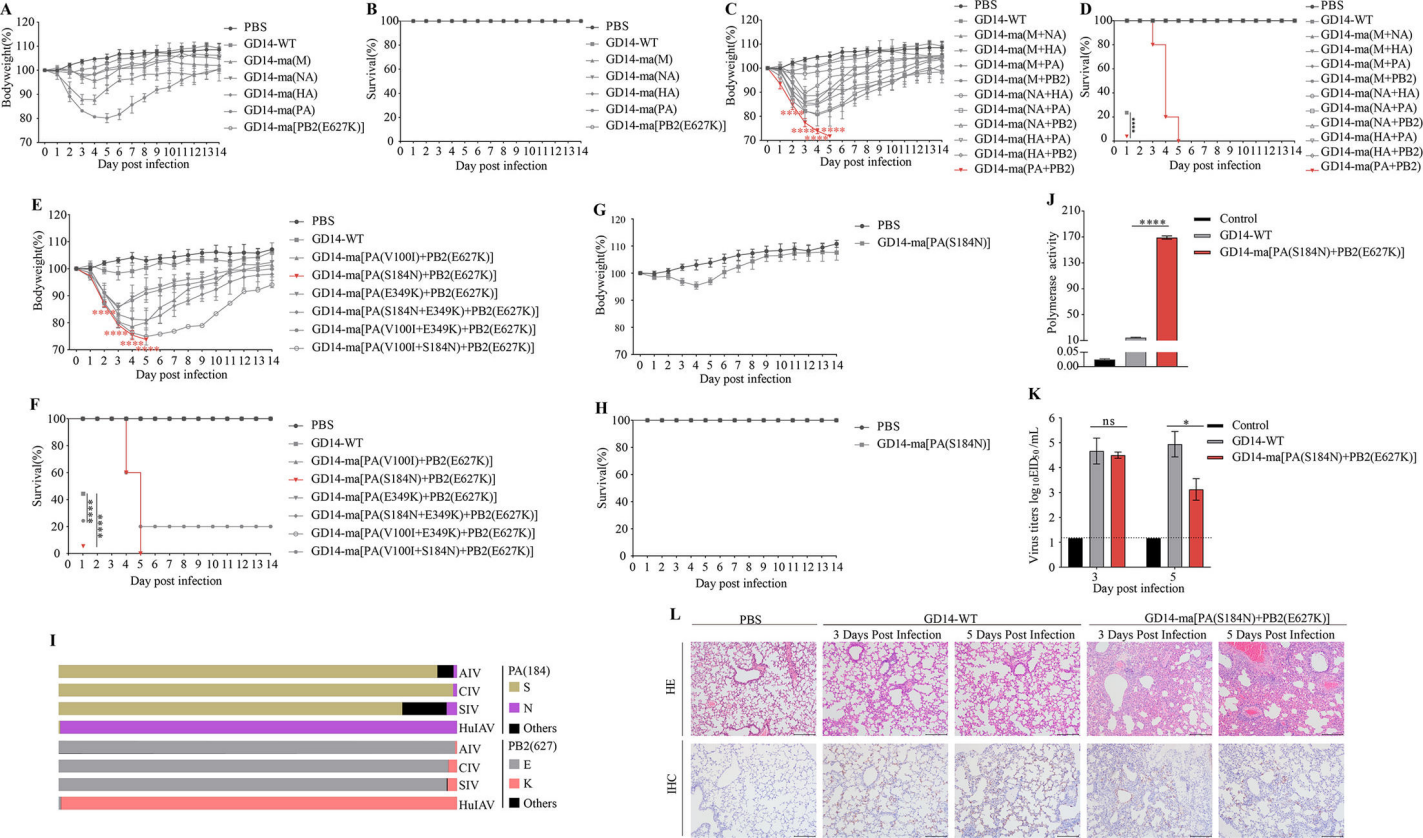
Synergistic lethal effects of PA (S184N) and PB2 (E627K) mutations in GD14-MA murine infections. (**A and B**) Body weight changes and survival rate of mice infected with single-segment replacement recombinant viruses. (**C and D**) Body weight changes and survival rate of mice infected with double-segment replacement recombinant viruses. (**E and F**) Body weight changes and survival rate of mice infected with double and triple-mutation recombinant viruses. (**G and H**) Body weight changes and survival rate of mice infected with GD14-ma[PA(S184N)] strains. (Five mice per group; mice were considered to have succumbed if they lost more than 25% of their initial body weight). (**I**) Prevalence of the PA (S184N) and PB2 (E627K) mutation across H3N2 subtypes of influenza A virus. (**J**) Polymerase activity, an empty plasmid vector devoid of the PA gene sequence was utilized as a negative control. (**K**) Lung viral titers at 3 and 5 dpi. The dashed line signifies the lower limit of detection. (**L**) Histological assessment of lung sections from experimental mice at 3 and 5 dpi via HE staining and IHC.

To identify the key mutations responsible for the lethality of the GD14-MA strain in mice, we constructed recombinant viruses by segment replacement using the GD14-WT strain as a genetic backbone. Mice that were infected with single-segment replacement strains GD14-ma(NA), GD14-ma(HA), and GD14-ma(PB2) showed no significant weight changes compared to GD14-WT over 14 dpi (Fig. 2A). By contrast, mice from the GD14-ma(M) and GD14-ma(PA) groups exhibited weight loss (Fig. 2A). Survival analysis revealed no mortality in any of the single-segment replacement groups (Fig. 2B). The double-segment replacement strains showed varied weight changes over 14 dpi (Fig. 2C). Notably, compared to the GD14-WT group, the GD14-ma(PA+PB2) group exhibited rapid weight loss within 5 days (*P* < 0.0001, Fig. 2C) and complete mortality for 5 dpi (*P* < 0.0001, Fig. 2D). These findings confirmed that the PA and PB2 segments are critical for lethality of the GD14-MA strain in mice.

To pinpoint the key mutations contributing to the lethality of GD14-ma(PA+PB2) in mice, we introduced specific mutations into the PA and PB2 segments and created double and triple mutation recombinant viruses. Compared to the GD14-WT group, mice infected with GD14-ma[PA(S184N)+PB2(E627K)] (*P* < 0.0001) and GD14-ma[PA(V100I+S184N)+PB2(E627K)] experienced rapid weight loss (Fig. 2E). Notably, all mice in the GD14-ma[PA(S184N)+PB2(E627K)] group had died by 5 dpi (*P* < 0.0001, Fig. 2F). These results preliminarily identified PA (S184N) and PB2 (E627K) as critical mutations for the lethality of the GD14-MA strain in mice, acting synergistically to induce death. To further confirm the synergistic effect of PA (S184N) and PB2 (E627K), we generated a single mutation virus strain GD14-ma[PA(S184N)] and intranasally inoculated 6-week-old female BALB/c mice at a titer of 10^6^ EID50 in 50 µL volumes. Compared to the PBS control group, these mice showed no significant weight changes (Fig. 2G) and no mortality was observed within 14 dpi (Fig. 2H), thereby confirming that the combined mutations PA (S184N) and PB2 (E627K) are essential for the lethal effects observed in mice infected with the GD14-MA strain.

We conducted an analysis of all PA and PB2 gene sequences of the H3N2 AIV, CIV, SIV, and HuIAV (Fig. 2I; Table S2). Our analysis revealed that residue 184 is predominantly S in AIV, CIV, and SIV, whereas N is the predominant residue in HuIAV. The prevalence of PA (184S) across these viruses was 95.11% in AIV (447 out of 470), 99.05% in CIV (312 out of 315), 86.29% in SIV (3398 out of 3938), and 0.36% in HuIAV (377 out of 105,697). By contrast, the prevalence of PA (184N) was significantly lower but still has been seen with this mutation, with 0.85% in AIV (4 out of 470), 0.95% in CIV (3 out of 315), 2.59% in SIV (102 out of 3938), and a notably higher prevalence of 99.63% in HuIAV (105,308 out of 105,697). Furthermore, we found that residue 627 is predominantly E in AIV, CIV, and SIV, whereas K is the predominant residue in HuIAV. The prevalence of PB2 (627E) was 99.54% in AIV (437 out of 439), 97.85% in CIV (318 out of 325), 97.48% in SIV (3822 out of 3921), and 0.51% in HuIAV (528 out of 102,685). Conversely, the prevalence of PB2 (627K) was considerably lower, with 0.46% in AIV (2 out of 439), 2.15% in CIV (7 out of 325), 2.37% in SIV (93 out of 3921), and a high prevalence of 99.44% in HuIAV (102,115 out of 102,685). Phylogenetic analysis of PA and PB2 gene segments from H3N2 AIV, CIV, SIV, and HuIAV revealed distinct evolutionary patterns. The phylogenetic trees demonstrated that PA and PB2 segments from each viral subtype formed separate clades with significant branch divergence, indicating substantial phylogenetic distance among these influenza strains (Fig.S1 and S2). Notably, polymerase activity assays demonstrated a marked increase in the GD14-ma[PA(S184N)+PB2(E627K)] strain when compared with the GD14-WT strain (*P* < 0.0001, Fig. 2J). Six-week-old female BALB/c mice were intranasally inoculated with 50 µL of GD14-WT or GD14-ma[PA(S184N)+PB2(E627K)] strains at 10^6^ EID50; control mice received 50 µL PBS. Lung tissues were collected from three mice per group at 3 and 5 dpi for viral titration and histopathological assessment. At 3 dpi, viral titers in the GD14-ma[PA(S184N)+PB2(E627K)] group did not differ significantly from the GD14-WT group. However, a significant decrease was observed at 5 dpi (*P* < 0.05, Fig. 2K). Histopathological assessments revealed that GD14-ma[PA(S184N)+PB2(E627K)] induced severe pneumonia, characterized by increased fibrinous exudates and inflammatory cell infiltration (Fig. 2L). Immunohistochemical detection of viral nucleoprotein confirmed that, in contrast to the GD14-WT group, the GD14-ma[PA(S184N)+PB2(E627K)] strain was not widely detected in the infected lungs at 5 dpi (Fig. 2L).

### Enhanced pathogenicity of GD14-ma[PA(V100I)+PB2(E627K)] strain in dogs

To evaluate the pathogenicity of GD14-WT, GD14-ma[PA(S184N)+PB2(E627K)], and GD14-MA strains in dogs, beagle dogs aged 9 to 12 weeks were inoculated via tracheal injection with 1 mL of 10^6^ EID50 of the respective strains (*n* = 6 per group), with a control group of four Beagles inoculated with 1 mL of PBS. Daily monitoring of body temperature and clinical symptoms was conducted over 8 dpi.

**Fig 3:**
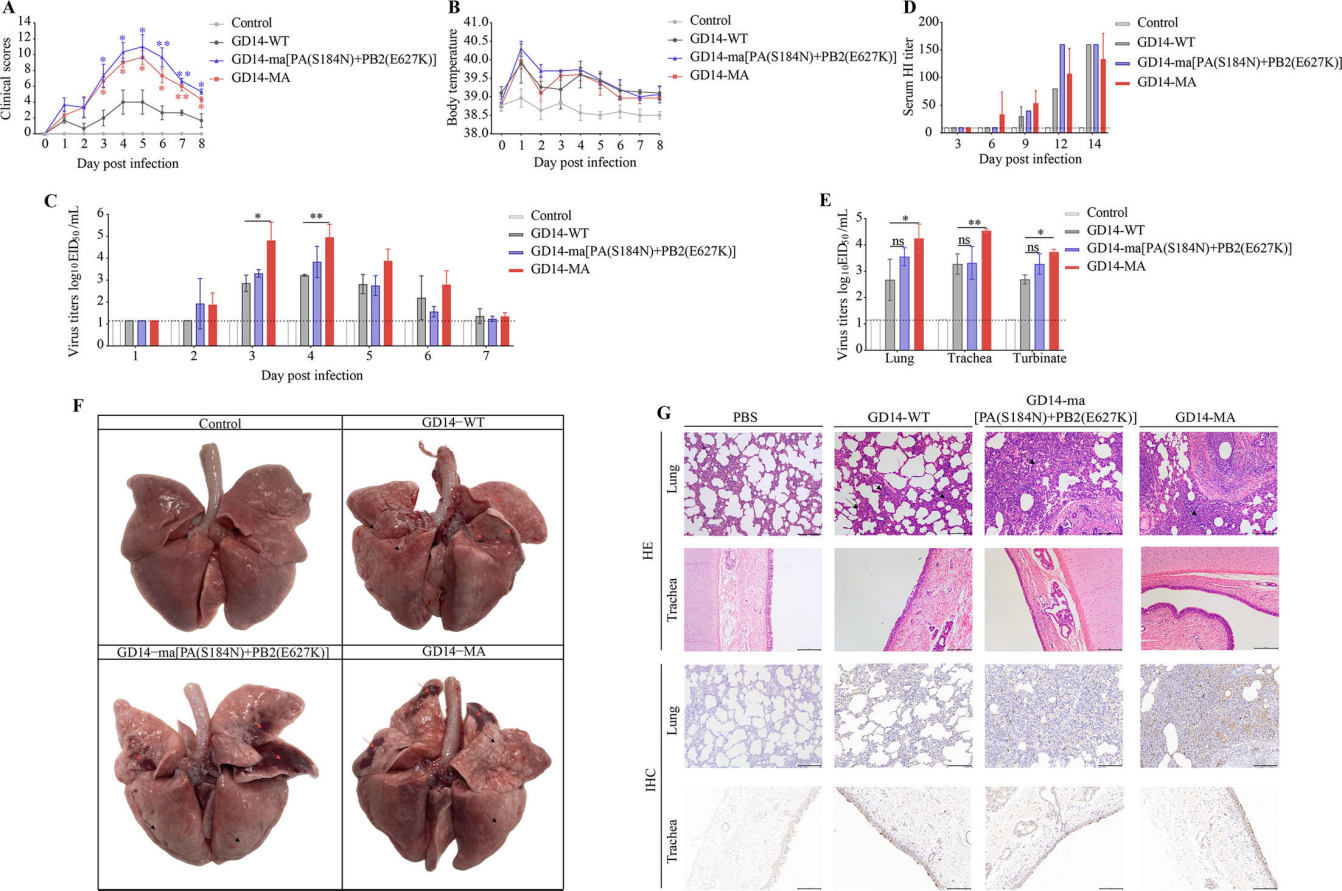
Enhanced pathogenicity of GD14-ma[PA(V100I)+PB2(E627K)] strain in dogs. (**A**) Clinical symptom scores in dogs. (**B**) Rectal body temperature measurements in dogs. (**C**) Viral titer in nasal swabs collected daily from 1 to 7 dpi. (**D**) Serum antibody titers in dogs. (**E**) Viral titers in the lungs, trachea, and turbinates at 3 dpi. (**F**) Gross appearance of the canine lungs. Black arrows delineate regions characteristic of pulmonary emphysema, whereas red arrows highlight areas indicative of hemorrhagic infiltration. (**G**) Histopathological examination of lung sections at 3 dpi using HE (the black arrow delineates the lesion site) staining and IHC.

Dogs inoculated with GD14-ma[PA(S184N)+PB2(E627K)] and GD14-MA strains exhibited significant clinical symptoms from 3 dpi, including reduced food intake, depression, tachypnea, mucous discharge, and coughing, whereas GD14-WT induced only mild symptoms. Clinical scores for GD14-ma[PA(S184N)+PB2(E627K)] and GD14-MA groups were significantly higher than for the GD14-WT group from 3 to 8 dpi (*P* < 0.05, Fig. 3A). Rectal temperatures indicated fevers (>39.5°C) in all infected groups from 3 to 5 dpi (Fig. 3B). Viral shedding, measured by nasal swab titers, was significantly higher in the GD14-MA group compared to the GD14-WT group at 3 (*P* < 0.05) and 4 dpi (*P* < 0.01), with no significant difference observed for the GD14-ma[PA(S184N)+PB2(E627K)] group (Fig. 3C). Hemagglutination inhibition (HI) assay results indicated that the GD14-MA group developed serum antibodies by 6 dpi, with no significant difference in serum antibody levels among the three infected groups at 9, 12, and 14 dpi (Fig. 3D). Infections with the GD14-MA strain resulted in markedly higher viral replication titers in the lung (*P* < 0.05), trachea (*P* < 0.01), and turbinate (*P* < 0.05) when compared to the GD14-WT strain, with no differences for the GD14-ma[PA(S184N)+PB2(E627K)] strain (Fig. 3E). Anatomical examination revealed localized multifocal emphysema and reddish-brown mottled areas in the lungs of dogs from the GD14-WT group, with more extensive hemorrhage and hardening in GD14-ma[PA(S184N)+PB2(E627K)] and GD14-MA groups (Fig. 3F). Histological evaluation showed mild interstitial cell proliferation in the lungs of dogs from the GD14-WT group, whereas GD14-ma[PA(S184N)+PB2(E627K)] and GD14-MA infections caused blurred bronchiolar alveolar spaces, substantial inflammatory exudation, and intravascular inflammatory cell clumps, primarily composed of lymphocytes, monocytes, and plasma cells (Fig. 3G). Compared to the GD14-WT group, the lungs of dogs infected with the GD14-MA strain exhibited widespread detection of virus-specific nucleoprotein antibodies. By contrast, antibodies specific to GD14-ma[PA(S184N)+PB2(E627K)] were not broadly present in the infected lungs (Fig. 3G).

### Evasion of host antiviral defenses by the GD14-ma[PA(S184N)+PB2(E627K)] strain

**Fig 4:**
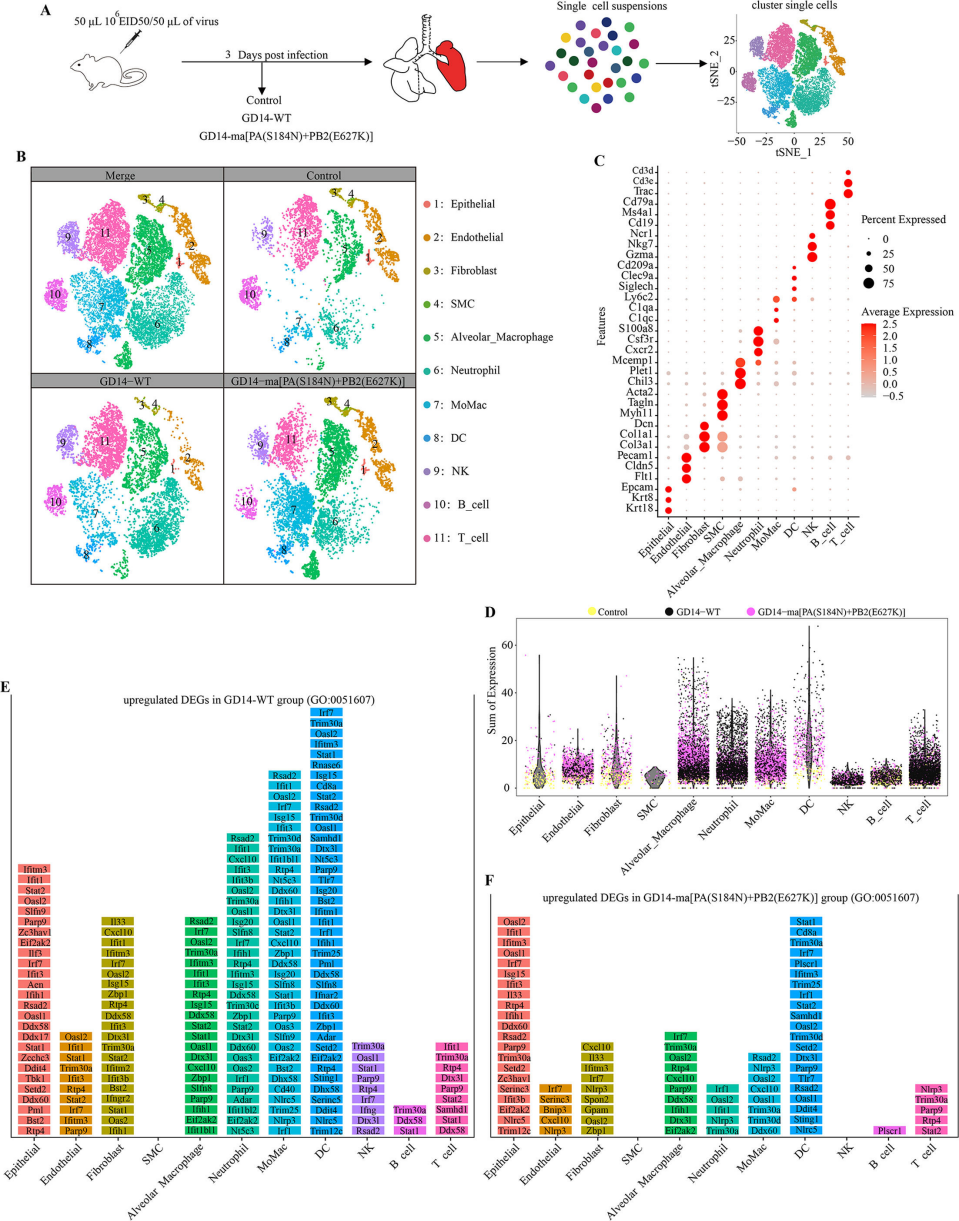
Evasion of host antiviral defenses by the GD14-ma[PA(S184N)+PB2(E627K)] strain. (**A**) Schematic overview of the study design. A single mouse per group was euthanized at 3 dpi, and individual samples were subjected to scRNA-seq analysis. (**B**) t-SNE plot illustrating the formation of 11 major clusters from 21,589 suspended lung cells. (**C**) Cell type annotation and dot plot. (**D**) Total UMI counts of host genes associated with the antiviral response (GO: 0051607) across different cell clusters (x-axis). Dots represent cells from various clusters, colored according to the samples. (**E, F**) Normalized expression of ranked DEGs. Histograms display significantly upregulated DEGs (log2 fold change ≥0.5, *P* ≤ 0.05) enriched in the “defense response to virus” (GO: 0051607) for each cell type. Genes are ranked from top to bottom based on their average expression levels in GD14-WT-infected cells (**E**) or GD14-ma[PA(S184N)+PB2(E627K)] infected cells (**F**) compared to control cells within each cell type.

To investigate the pathogenicity discrepancies between the GD14-ma[PA(S184N)+PB2(E627K)] and GD14-WT strains in mice, we collected left lung cells from mice at 3 dpi following exposure to GD14-WT and GD14-ma[PA(S184N)+PB2(E627K)] or PBS (Fig. 4A). Employing the BD Rhapsody platform, we performed single-cell RNA sequencing (scRNA-seq) to delineate the transcriptomic profiles, ensuring sequencing quality (Table S3). The analysis encompassed 21,589 cells (Fig. and Table S4), discerning 11 distinct clusters via t-SNE (Fig. 4B). Heatmaps illustrated the expression patterns of signature genes within each cluster, identifying non-immune clusters of epithelial cells, endothelial cells, fibroblast cells, and smooth muscle cells (SMC), as well as immune clusters of alveolar macrophages, neutrophils, monocytes and mononuclear macrophages (MoMac), dendritic cells (DC), natural killer cells (NK), B cells, and T cells (Fig. 4C; Table S5).

Differentially expressed genes (DEGs) revealed substantial transcriptional changes in the lung tissues of mice infected with GD14-ma[PA(S184N)+PB2(E627K)] and GD14-WT strains compared to control (Fig. S4). We further examined the expression of genes associated with the “viral defense response” (GO: 0051607) and identified an upregulation of antiviral genes in various cell types in both infected groups (Fig. 4D). Specifically, we pinpointed upregulated differential antiviral genes in each cell type using stringent criteria (Log2FC > 0.5, *P* < 0.05), ranking them by their average expression within clusters (Fig. 4E and F). The GD14-WT group exhibited 74 upregulated DEGs associated with the antiviral response, surpassing the 64 identified in the GD14-ma[PA(S184N)+PB2(E627K)] group (Table S6). Within the GD14-WT group, the highest expression of antiviral-related genes was observed in epithelial cells, fibroblast cells, alveolar macrophages, neutrophils, MoMac, and DC. By contrast, within the GD14-ma[PA(S184N)+PB2(E627K)] group, epithelial cells and DC were the predominant contributors to the expression of antiviral-related genes. Comparative analysis indicated that GD14-ma[PA(S184N)+PB2(E627K)] significantly downregulated antiviral-related genes in all cellular clusters except SMC, compared to the GD14-WT group.

### Elevated inflammatory responses and cytokine storms elicited by the GD14-ma[PA(S184N)+PB2(E627K)] strain in murine pulmonary infection

**Fig 5:**
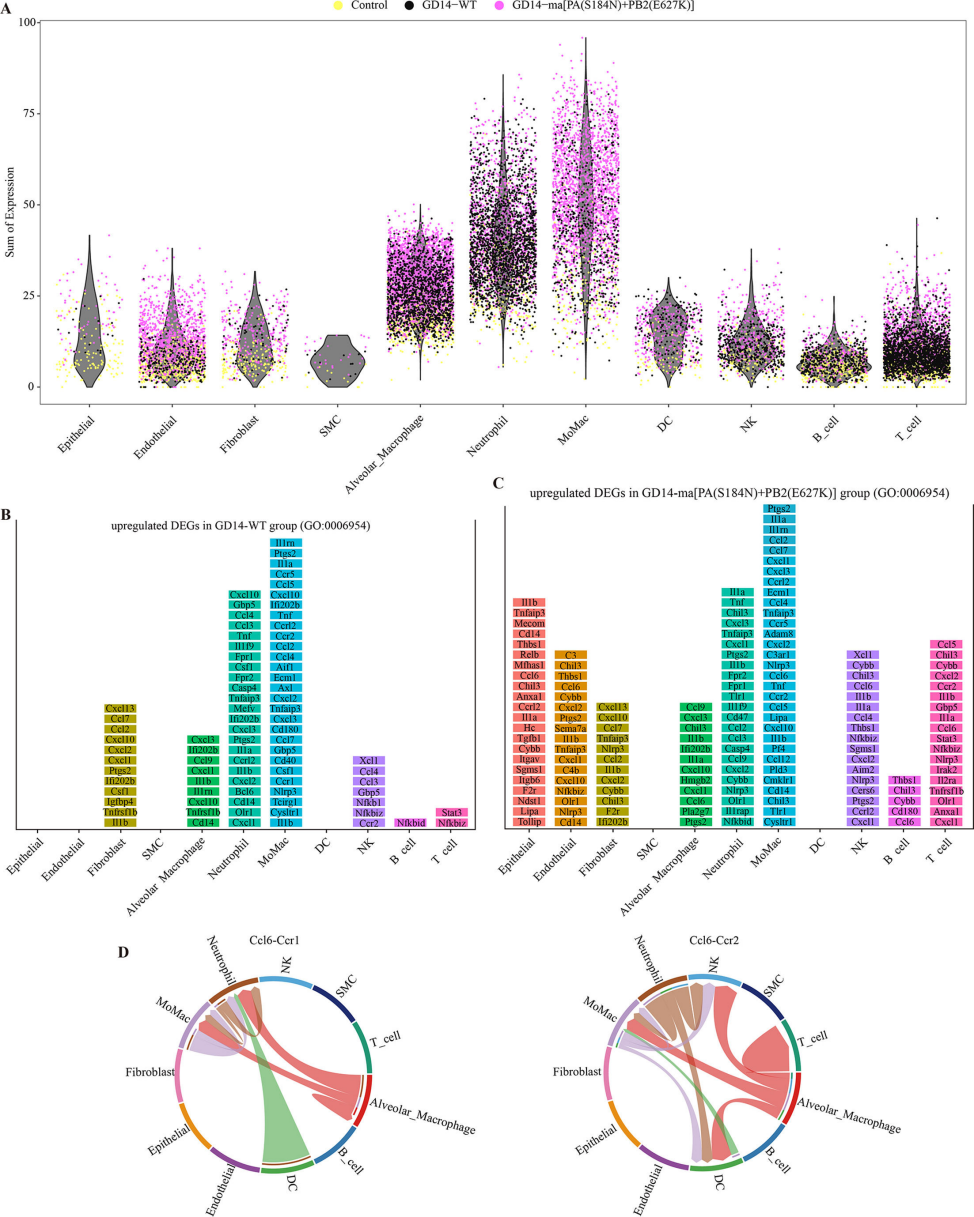
Elevated inflammatory responses and cytokine storms elicited by the GD14-ma[PA(S184N)+PB2(E627K)] strain in murine pulmonary infection. (**A**) UMI counts of host genes related to the “inflammatory response” (GO: 0006954) are shown across different cell clusters on the x-axis. Dots represent individual cells from various clusters, differentiated by color according to their sample groups. (**B, C**) Normalized expression levels of highly ranked DEGs. Histograms illustrate significantly upregulated DEGs (log2 fold change ≥0.5, *P* ≤ 0.05) that are enriched for the “inflammatory response” (GO: 0006954) in each cell type. The genes are ranked from top to bottom based on their average expression levels in GD14-WT-infected cells (**B**) or GD14-ma[PA(S184N)+PB2(E627K)]-infected cells (**C**) relative to control cells within the same cell type. (**D**) Predicted interaction map depicting the communication network mediated by CCL6 following challenge with GD14-ma[PA(S184N)+PB2(E627K)] strain.

Overzealous immune reactions can induce a cytokine storm, potentially causing tissue damage, organ failure, and host mortality. Consequently, we conducted a detailed analysis of DEGs linked to the “inflammatory response” (GO: 0006954) across all cell clusters. Our findings indicated that alveolar macrophages, neutrophils, and MoMac populations were prolific sources of inflammatory mediators (Fig. 5A). We detailed the upregulated inflammatory genes in each cell type for both the GD14-WT and GD14-ma[PA(S184N)+PB2(E627K)] groups, ranked by their average expression levels (Log2FC > 0.5, *P* < 0.05) within the respective cell clusters (Fig. 5B and C). The GD14-ma[PA(S184N)+PB2(E627K)] group displayed 111 upregulated inflammatory genes, surpassing the 87 observed in the GD14-WT group (Table S7). MoMac and neutrophils were the primary sources of inflammatory factors in the GD14-WT group, while the GD14-ma[PA(S184N)+PB2(E627K)] group revealed significant contributions from epithelial cells, endothelial cells, neutrophils, and MoMac. Notably, the GD14-ma[PA(S184N)+PB2(E627K)] group exhibited a marked increase in both the variety and quantity of inflammatory factors in epithelial cells, NK cells, T cells, and endothelial cells, as well as an elevated total inflammatory factor content per cell within MoMac clusters. The chemokine CCL6 was specifically upregulated in various cell types in the GD14-ma[PA(S184N)+PB2(E627K)] group, including epithelial cells, endothelial cells, fibroblasts, alveolar macrophages, MoMac, NK cells, B cells, and T cells. Utilizing CellChat, we illustrated the potential communication network involving CCL6 and its receptors among different cell populations (Fig. 5D).

### Host cell infection patterns and viral transcript expression in murine lung tissue infected with CIV at single-cell resolution

**Fig 6:**
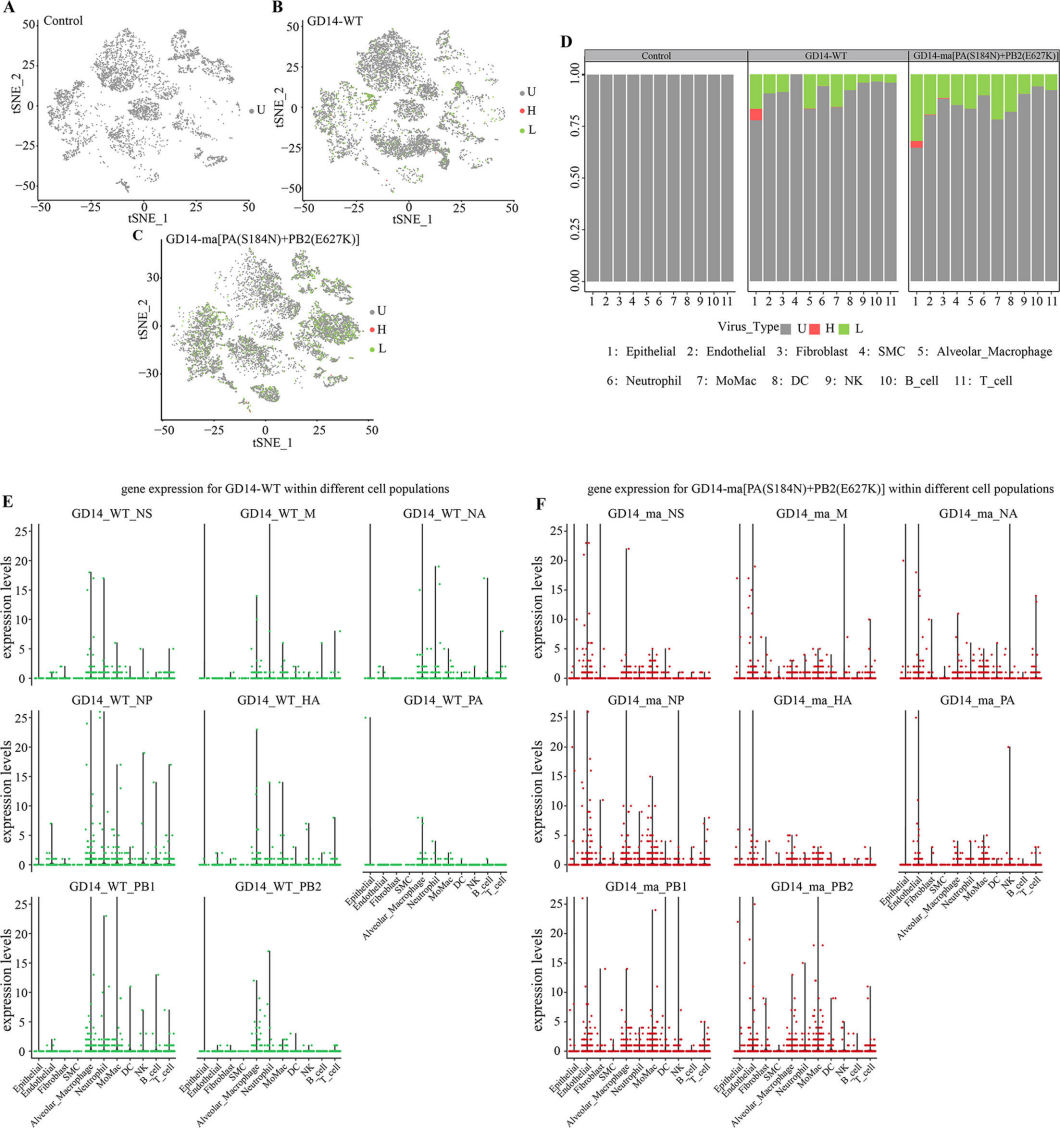
Host cell infection patterns and viral transcript expression in murine lung tissue infected with CIV at single-cell resolution. CIV-susceptible cells were classified into highly infected (H, total UMI count ≥8 with expression of each viral segment), lowly infected (L, total UMI count ≥1), and undetected (U, UMI count = 0) based on CIV transcript expression counts. (**A–C**) T-SNE plots illustrate the expression of viral genes at the single-cell level. (**D**) The y-axis displays the percentages of cells susceptible to CIV infection, categorized as highly infected, lowly infected, and undetected cells. (**E, F**) Violin plots represent the distribution of gene expression levels for GD14-WT (**E**) and GD14-ma[PA(S184N)+PB2(E627K)] (**F**) within different cell populations, with the y-axis indicating the relative expression levels.

We identified CIV-infected host cells by tracking intracellular segmented mRNAs at single-cell resolution. CIV-susceptible cells were classified into highly infected (H, total UMI count ≥8 with expression of each viral segment), lowly infected (L, total UMI count ≥1), and undetected (U, UMI count = 0) based on CIV transcript expression counts. Figure 6A through C illustrates viral gene expression at the single-cell level, with viral genes detected in samples infected with GD14-WT and GD14-ma[PA(S184N)+PB2(E627K)] strains, but not in control samples (Fig. 6A through C; Table S8). The percentages of highly infected, lowly infected, and undetected cells are depicted on the y-axis. The GD14-ma[PA(S184N)+PB2(E627K)] group showed a slightly higher proportion of lowly infected cells compared to the GD14-WT group (Fig. 6D). There was no difference in the proportion of cells with high viral load between GD14-WT and GD14-ma[PA(S184N)+PB2(E627K)] strains. Both strains exhibited high viral loads in epithelial cells. GD14-ma[PA(S184N)+PB2(E627K)] additionally showed high loads in endothelial cells and fibroblast cells, while the GD14-WT strain demonstrated high loads in alveolar macrophages, neutrophils, and MoMac. Violin plots indicate high expression of eight viral genes in epithelial cells, alveolar macrophages, and neutrophils for GD14-WT (Fig. 6E), and in epithelial and endothelial cells for GD14-ma[PA(S184N)+PB2(E627K)] (Fig. 6F).

## DISCUSSION

In this study, we successfully established a lethal mouse model for H3N2 CIV and isolated a lethal mouse strain, designated as GD14-MA. Employing reverse genetics, we identified the PA (S184N) and PB2 (E627K) mutations as critical factors contributing to lethality in mice. Pathogenicity studies in dogs revealed that the GD14-ma[PA(S184N)+PB2(E627K)] strain exhibited significantly enhanced virulence compared to the GD14-WT strain. Single-cell RNA sequencing of infected mouse lung tissues showed that GD14-ma[PA(S184N)+PB2(E627K)] effectively evaded host antiviral responses, inducing a robust inflammatory reaction.

To elucidate the mechanism underlying the enhanced pathogenicity of the GD14-ma[PA(S184N)+PB2(E627K)] strain, we assessed its polymerase activity and viral replication titers in mice and dogs. Compared to the GD14-WT strain, the polymerase activity of the GD14-ma[PA(S184N)+PB2(E627K)] strain was significantly enhanced. However, this did not result in increased viral replication in mice and dogs. This indicates that the GD14-ma[PA(S184N)+PB2(E627K)] strain does not enhance its pathogenicity by increasing its replication titer.

The high mutation rate of the IAV genome serves as the genetic basis for its changes in pathogenicity and cross-species transmission, with the PB2 E627K mutation being extensively characterized. Previous studies have demonstrated that the PB2 E627K mutation enhances the polymerase activity of H3N8, H5N1, and H7N9 AIVs isolated from humans at lower temperatures (37–40). The PB2 E627K mutation has also been shown to increase the pathogenicity of H4N6, H5N6, H5N8, H6N1, H7N7, H7N9, and H9N2 AIVs in mice (38, 41–46). However, the PB2 E627K mutation does not universally enhance the pathogenicity of IAVs in mice. Research has indicated that the PB2 E627K mutation attenuates the virulence of IAV containing the 2009 H1N1 pandemic polymerase proteins in mice (47). Moreover, our previous studies have shown that the PB2 E627K mutation does not enhance the pathogenicity of CIV in mice and dogs, which is consistent with the findings of the present study (48). Therefore, the impact of the PB2 E627K mutation on the mammalian adaptation and pathogenicity of IAVs is diverse.

In addition, the mutation frequencies of PA (S184N) and PB2 (E627K) exhibited a gradual increase across AIV, CIV, SIV, and HuIAV, with both PA (S184N) and PB2 (E627K) being present at rates higher than 99% in HuIAV (Fig. 2I; Table S2). However, phylogenetic analysis revealed significant genetic divergence in the PA and PB2 genes among H3N2 AIV, CIV, SIV, and HuIAV. These mutations may be subject to host-specific selective pressures and influenced by the internal genetic composition of the virus, potentially representing stochastic events in the evolutionary history of IAVs. Nevertheless, the relatively high prevalence of the PA (S184N) mutation, which was first identified in this study, in HuIAV suggests that this mutation may have a significant impact on the biology and pathogenesis of IAV. Therefore, further investigation into the functional and phenotypic consequences of the PA (S184N) mutation is warranted.

Advancements in scRNA-seq have enabled a detailed exploration of the murine response to CIV infection, shedding light on the mechanisms underlying the enhanced pathogenicity of the GD14-ma[PA(S184N)+PB2(E627K)] strain. In the murine lung, we identified 11 distinct cellular clusters and conducted a comprehensive differential gene expression analysis across these clusters (Fig. 4B). In addition, we confirmed the infection status of individual cells with H3N2 CIV. The proportion of cells with high viral loads in the lungs of mice infected with GD14-WT and GD14-ma[PA(S184N)+PB2(E627K)] strains showed no significant difference (Fig. 6D), which is consistent with the viral titers observed in the lungs at 3 dpi (Fig. 2K). This observation further substantiates that the GD14-ma[PA(S184N)+PB2(E627K)] strain does not augment its virulence in mice by increasing viral titers. DEG analysis disclosed a significant suppression of the antiviral response in all cell clusters, excluding SMC, upon GD14-ma[PA(S184N)+PB2(E627K)] infection compared to GD14-WT (Fig. 4E and F). This observation implies that the GD14-ma[PA(S184N)+PB2(E627K)] strain has evolved a markedly enhanced ability to evade the host’s antiviral defenses, which could account for the elevated pathogenicity associated with this mutant strain. Excessive inflammatory responses can lead to a cytokine storm, exacerbating mouse mortality. We found that GD14-ma[PA(S184N)+PB2(E627K)] significantly upregulated the expression of inflammatory genes in various lung cell clusters compared to GD14-WT, particularly in epithelial, endothelial cells, and MoMac clusters (Fig. 5B and C). Notably, the chemokine CCL6 was specifically upregulated in response to GD14-ma[PA(S184N)+PB2(E627K)], suggesting a role in the inflammatory response and potential contribution to pulmonary inflammation. The intercellular communication network is crucial in the cytokine storm and pneumonia induced by IAV (49). Our prediction of the intercellular communication network involving CCL6 revealed extensive ligand-receptor interactions across various cell populations (Fig. 5D), which may lead to inflammatory lung injury. The molecular mechanisms facilitating the intercellular communication among diverse cellular clusters, which induce the release of inflammatory cytokines, warrant further investigation.

Traditionally, avian-origin low pathogenicity influenza viruses require multiple serial passages in mouse lungs to adapt and become virulent in the murine model. In this study, to rapidly establish a lethal mouse model for H3N2 CIV, we initiated with the GD14-WT strain and, by altering the age of mice at each passage, successfully adapted a highly virulent strain, GD14-MA, after only 18 passages, as evidenced by rapid weight loss and complete mortality by 5 dpi. This method of alternating passages in mice of different ages accelerated the development of the CIV H3N2 lethal mouse model, offering an innovative approach for the construction of mouse-adapted models of low-pathogenicity IAVs.

## MATERIALS AND METHODS

### Viruses and cells

Human embryonic kidney (HEK) 293T cells and Madin-Darby canine kidney (MDCK) cells are maintained in a 5% CO2 at 37°C, in Dulbecco’s modified Eagle’s medium (DMEM, Gibco, C11995500BT) supplemented with 10% fetal bovine serum (FBS, Gibco, A5669701) and 1× penicillin-streptomycin (Gibco, 15140-122). The H3N2 CIV (GD14-WT) is preserved in our laboratory.

### Establishment of a lethal H3N2 CIV infection model in mice

Four-week-old female BALB/c mice (n=5 per group) were intranasally inoculated with 50 μL of the GD14-WT strain at a titer of 10^6^ 50% egg infectious doses (EID50). At 5 dpi, the mice were euthanized under deep anesthesia, and their lungs were aseptically excised. Lung tissues were subjected to homogenization at −20°C, followed by centrifugation at 12,000 × *g* for 10 minutes at 4°C. The resulting supernatant was filtered through a 0.45 μm pore-sized membrane filter (BIOFIL, FPE404013) to achieve sterility, and this filtrate was designated as the first passage (P1).

Subsequently, 50 μL of the P1 supernatant was used to intranasally inoculate another cohort of 4-week-old female BALB/c mice, and this process was serially repeated until the sixth passage was achieved. Thereafter, 50 μL of the sixth-passage supernatant was used to inoculate 5-week-old female BALB/c mice, progressing the passage to the 13th generation. The 13th-passage supernatant was then employed to intranasally inoculate 6-week-old female BALB/c mice, thereby extending the passage to the 18th generation (P18).

The 18th-passage mouse-adapted strain was further purified through plaque assay in MDCK cells to ensure clonal purity. Ultimately, 6-week-old female BALB/c mice were inoculated intranasally with 50 μL of a 10^6^ EID50 suspension of the GD14-P18A, GD14-P18B, GD14-P18C, and GD14-WT strains. Control mice received an equivalent volume of phosphate-buffered saline (PBS) via intranasal administration. Body weight and survival rates were closely monitored for 14 dpi. At 3 and 5 dpi, three mice were humanely euthanized to harvest lung tissues for viral titration. Lung sections were prepared for subsequent histopathological and immunohistochemical examination. The aforementioned mouse experiments were conducted with the approval of the Experimental Animal Welfare Ethics Committee of South China Agricultural University (approval number: 2022c007).

### Identification of critical lethal mutations of the GD14-MA strain in mice

To elucidate the virulence factors of the GD14-MA strain, we generated recombinant viruses by replacing the Matrix (M), Neuraminidase (NA), Hemagglutinin (HA), Polymerase Acidic (PA), and Polymerase Basic 2 (PB2) gene segments of GD14-WT with those from GD14-MA using established reverse genetics for IAV (50). The single-segment reassortant viruses were designated as GD14-ma(M), GD14-ma(NA), GD14-ma(HA), GD14-ma(PA), and GD14-ma(PB2), respectively. Furthermore, double-segment reassortant viruses were termed GD14-ma(M+NA) to GD14-ma(PA+PB2). These viruses were administered intranasally to 6-week-old female BALB/c mice at 10^6^ EID50 in 50 μL volumes, with daily monitoring of body weight and survival rate. We identified a synergistic effect between the PA and PB2 segments in enhancing virulence. Specific mutations in these segments were introduced to create GD14-ma[PA(V100I)+PB2(E627K)] to GD14-ma[PA(V100I+S184N)+PB2(E627K)]. These mutants were inoculated into mice and observed for 14 dpi for body weight and survival rate. To evaluate the individual contributions of the PA (S184N) and PB2 (E627K) mutations, we generated a single mutation virus strain, GD14-ma[PA(S184N)], and assessed its pathogenicity in mice. To further elucidate the differential pathogenicity induced by the GD14-ma[PA(V100I)+PB2(E627K)] and GD14-WT strains, lung tissues from three mice per group were collected at 3 and 5 dpi for viral titration and histopathological examination.

### Pathogenicity of GD14-WT, GD14-ma[PA(V100I)+PB2(E627K)] and GD14-MA in dogs

In this study, we utilized beagle dogs aged 9–12 weeks, ensuring they were free of H3N2 CIV infection through polymerase chain reaction and serological assays. Three experimental groups, each comprising six dogs, were anesthetized and inoculated via tracheal injection with 1 mL of 10^6^ EID50 of GD14-WT, GD14-MA, or GD14-ma[PA(S184N)+PB2(E627K)] strains. A control group of four Beagles received 1 mL of PBS. Clinical symptoms were monitored up to 8 dpi, scored as per the criteria outlined in Table S1. Nasal swabs were daily collected, and serum samples were obtained from day 3 to 14 dpi. At 3 dpi, three dogs from each challenge group and one control dog were euthanized via intravenous pentobarbital injection, followed by necropsy. Lungs, tracheas, and turbinates were harvested to evaluate viral replication, with lung and tracheal sections prepared for histopathological and immunohistochemical analyses.

### Polymerase activity

We combined 100 ng each of the PA, Polymerase Basic ^1^(PB1), PB2, and Nucleoprotein (NP) plasmids with a CIV luciferase reporter plasmid (100 ng) and a renilla luciferase reporter plasmid (pRL-TK, 10 ng). The plasmid mix was then transfected into 293T cells using Lipofectamine 3000 (ThermoFisher, L3000015) and incubated at 37°C for 24 hours. Luciferase production was measured according to the manufacturer’s instructions, utilizing a dual-luciferase reporter assay system (Promega, E1910).

### Sequence alignment and phylogenetic analysis

All PA and PB2 sequences of the H3N2 subtype of AIV, CIV, swine influenza A virus (SIV), and human influenza A virus (HuIAV) were downloaded from the Global Initiative on Sharing All Influenza Data (GISAID). Subsequently, sequence alignment was performed using the multiple sequence alignment software mafft. A statistical analysis was conducted on the position 184 of the PA protein and the position 627 of the PB2 protein across different H3N2 influenza subtypes to ascertain the prevalence of PA (S184N) and PB2 (E627K) in these subtypes. Subsequently, we conducted the phylogenetic analysis using MEGA X software and constructed the phylogenetic tree with the Neighbor-joining method. The reliability of the tree topology was evaluated by bootstrap analysis with 1,000 replicates.

### Single-cell RNA sequencing

At 3 dpi, left lung cellular specimens were obtained from mice inoculated with GD14-WT, GD14-ma[PA(S184N)+PB2(E627K)], or PBS. The BD Rhapsody system was utilized for single-cell transcriptomic profiling, employing a limited dilution strategy to disperse cells into microwells for barcoded bead pairing. Following cell lysis, mRNA was hybridized to barcoded oligos, and cDNA was synthesized, tagged with a Total Unique Molecular Identifier (UMI) and cell barcode. Libraries were prepared using the BD Rhapsody WTA workflow and quantified with Agilent and Qubit assays, followed by 150 bp paired-end sequencing on a DNBSEQ-T7 Sequencer.

### Single-cell RNA statistical analysis

Data processing involved fastp for adaptor sequence filtering and quality read removal (51). Gene expression was quantified with STARsolo using the mouse genome mm10, cells with over 200 genes and less than 20% mitochondrial UMI rate were included post-filtering. The Seurat package (version: 4.1.1) normalized and scaled the data based on UMI counts and mitochondrial rates. Principal component analysis (PCA) was performed on the top 2000 variable genes, with the top 10 components used for t-Distributed Stochastic Neighbor Embedding (t-SNE).

### Differential gene expression and viral burden assessment across cellular compartments and cell communication dynamics

To discern differential gene expression across samples, the FindMarkers function employing the Wilcoxon rank sum test was applied with the following criteria: 1. Log2 fold change (Log2FC)>0.25; 2. *p*<0.05; 3. minimum percentage (min.pct)>0.1. Host cells infected with CIV were ascertained by monitoring the fragmented mRNAs of CIV at the single-cell level. Cells exhibiting robust CIV gene expression—characterized by a minimum of one transcript per gene—were classified as highly infected. Accordingly, cells within clusters permissive to CIV were stratified into highly infected cells (H, UMI counts of viral transcripts ≥ 8 with expression of each viral segment), lowly infected cells (L, UMI counts of viral transcripts ≥ 1), and undetected cells (U, UMI counts of viral transcripts = 0). To enable a systematic analysis of cell–cell communication molecules, we applied cell communication analysis based on CellChat, a public repository of ligands, receptors, and their interactions (52).

### Statistical analysis

Statistical analyses were performed using GraphPad Prism version 9.0 software. Results are expressed as the mean ± standard deviation (SD). Group comparisons were evaluated using Student’s t-test. Statistical significance was determined at *P* > 0.05 (non-significant, ns), *P* < 0.05 (*), *P* < 0.01 (**), *P* < 0.001 (***), and *P* < 0.0001 (****). Animal survival data were analyzed employing the Kaplan-Meier method to ascertain differences among groups.

## Data Availability

The data are all displayed in the published article.

## SUPPLEMENTAL MATERIAL

Document S1 jvi.01984-24-s0001.docx Figures S1 to S4 and Tables S1 to S4.

Table S5 jvi.01984-24-s0002.xlsx Expression of marker genes within each cellular cluster.

Table S6 jvi.01984-24-s0003.xlsx Upregulated DEGs associated with the "viral defense response" (GO: 0051607) across all cellular clusters in the GD14-WT and GD14-ma[PA(S184N)+PB2(E627K)] groups.

Table S7 jvi.01984-24-s0004.xlsx Upregulated DEGs associated with the "inflammatory response" (GO: 0006954) across all cellular clusters in the GD14-WT and GD14-ma[PA(S184N)+PB2(E627K)] groups.

Table S8 jvi.01984-24-s0005.xlsx Infection status of CIV in all cellular clusters.

ASM does not own the copyrights to Supplemental Material that may be linked to, or accessed through, an article. The authors have granted ASM a non-exclusive, world-wide license to publish the Supplemental Material files. Please contact the corresponding author directly for reuse.
