# Supplementary material for: Synergistic effects of PA (S184N) and PB2 (E627K) mutations on the increased pathogenicity of H3N2 canine influenza virus infections in mice and dogs
Source: J Virol. 2025 Apr 4;99(5):e01984-24. doi: 10.1128/jvi.01984-24 (PMC12090714; doi:10.1128/jvi.01984-24)
Supplement: Document S1 — Figures S1 to S4 and Tables S1 to S4. [file jvi.01984-24-s0001.docx]

**Supplemental information**

Synergistic Effects of PA (S184N) and PB2 (E627K) Mutations on the Increased Pathogenicity of H3N2 Canine Influenza Virus Infections in Mice and Dogs

Xiangyu Xiao^1^, Xinrui Wang^1^, Fengpei Xu^1^, Yanting Liang^1^, Yi Luo^1^, Shoujun Li^1,#^, Pei Zhou^1,#^

**^
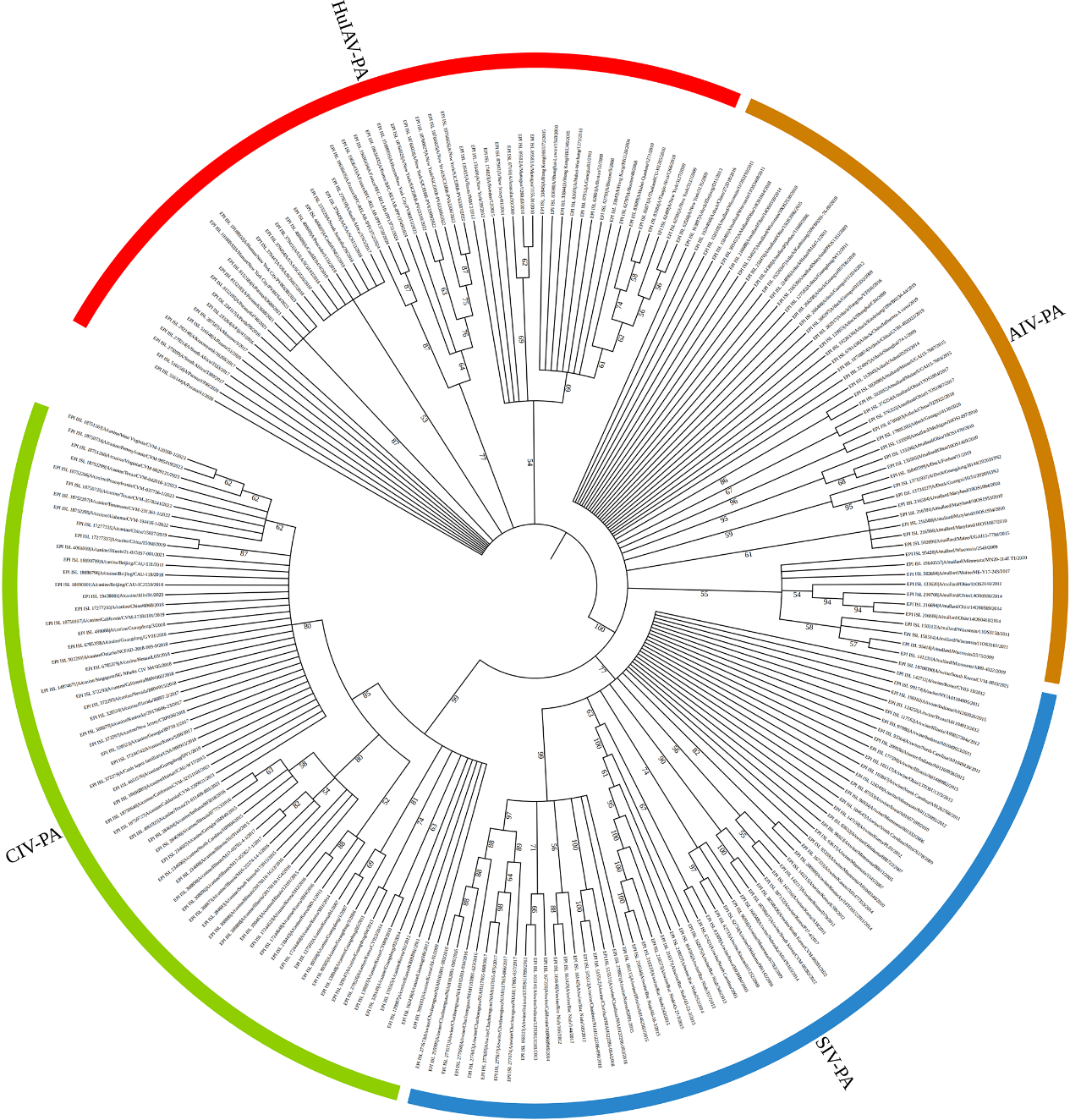
^**

**Figure S1** Phylogenetic tree of PA segments from the H3N2 AIV, CIV, SIV and HuIAV.

**^
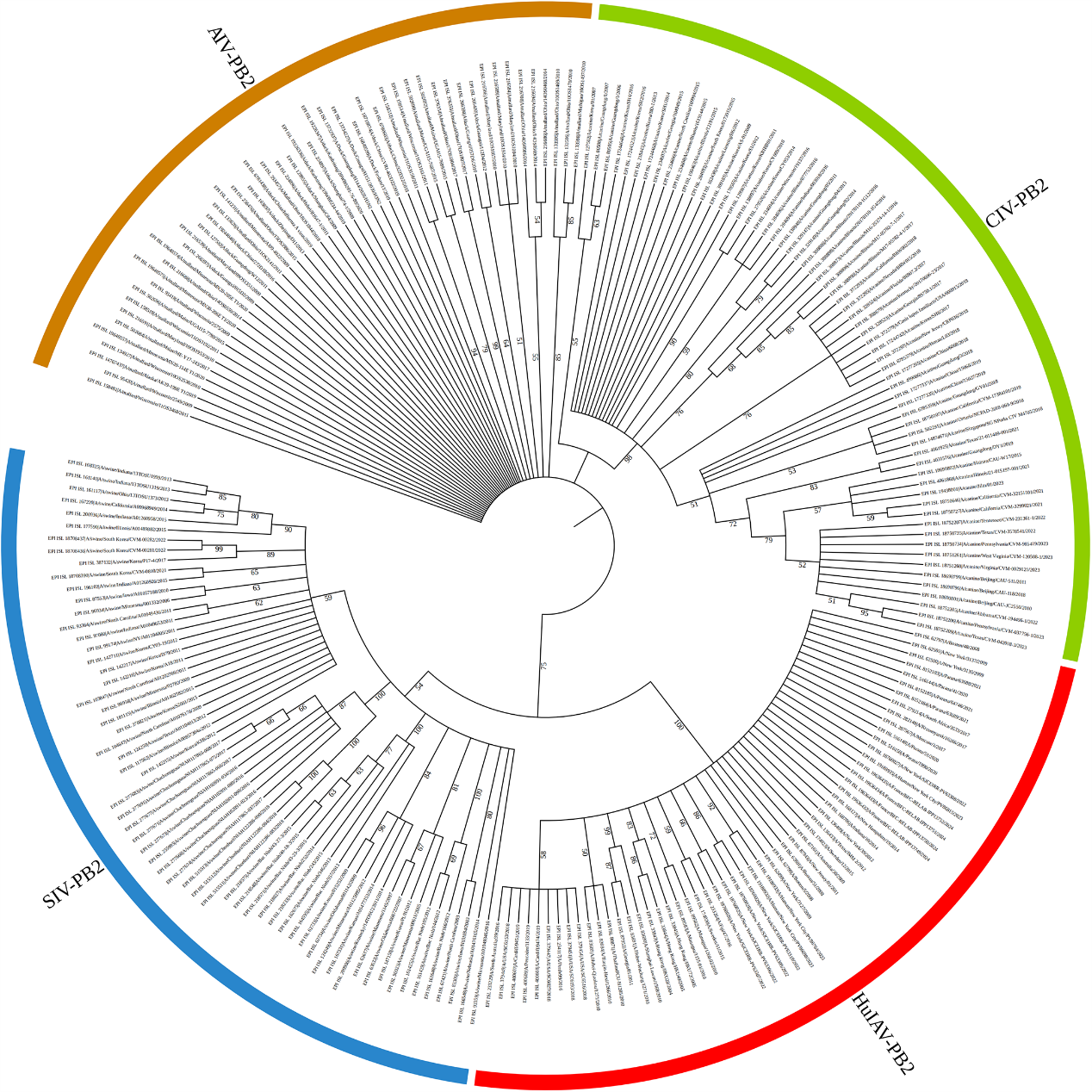
^**

**Figure S2** Phylogenetic tree of PB2 segments from the H3N2 AIV, CIV, SIV and HuIAV.

**
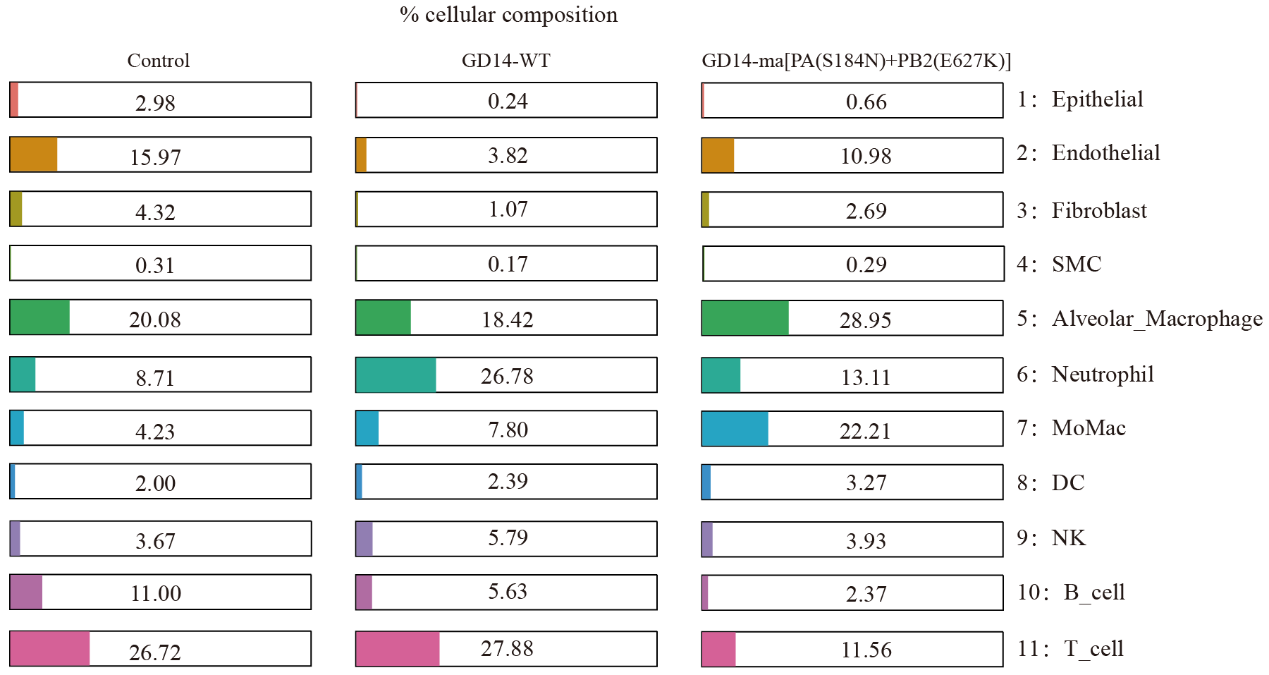
Figure S3** Distribution and proportions of cellular subtypes across experimental groups.

**
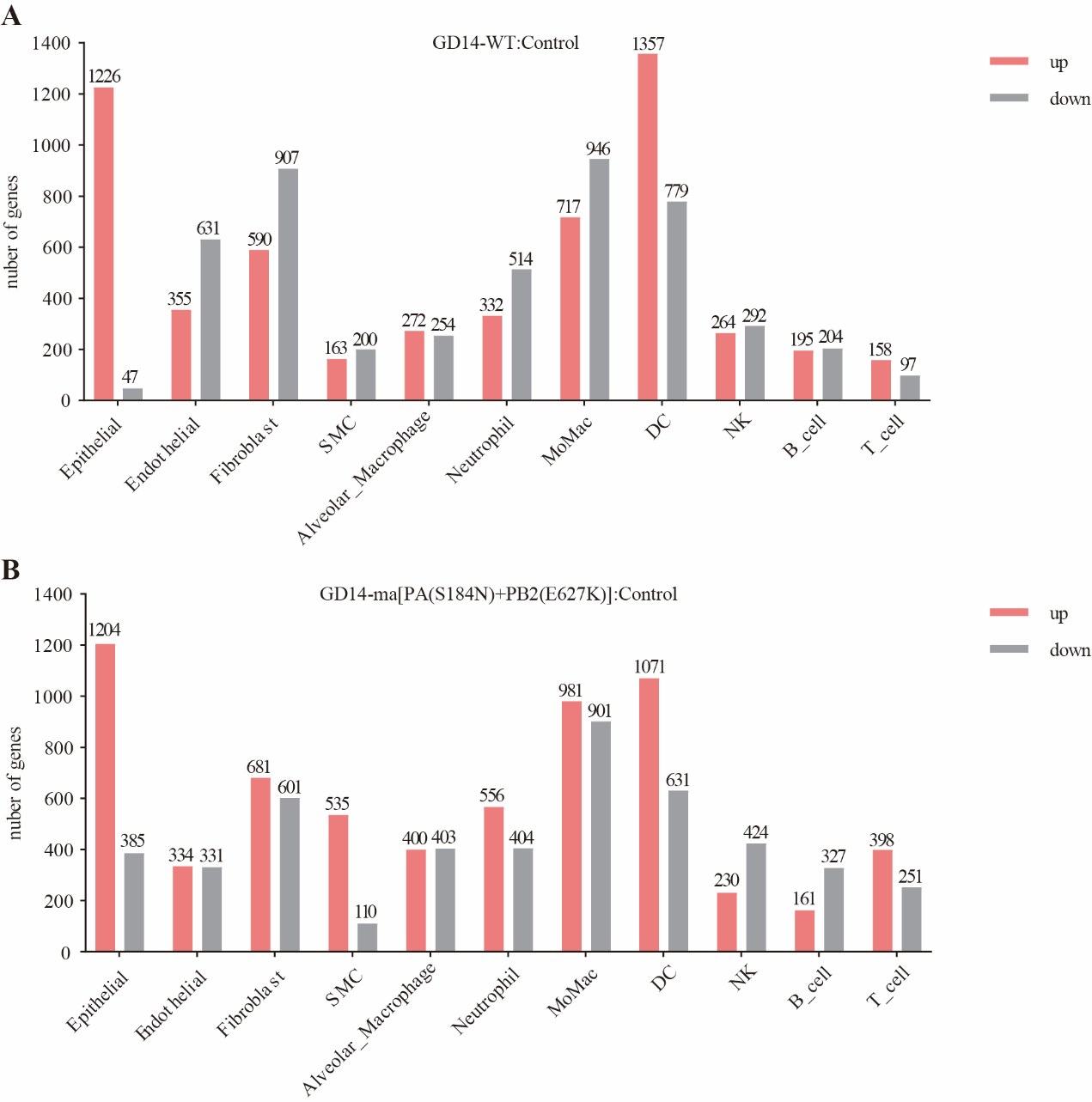
Figure S4** DEGs among cellular clusters in GD14-WT and GD14-ma[PA(S184N)+PB2(E627K)] groups.

**Table S1** Canine clinical symptomatology scoring system.

| Scoring Item | Scoring Criteria | | |
| --- | --- | --- | --- |
|  | 0 | 1 | 2 |
| Mental State | Normal mentation, alert with bright eyes, and agile movements | Mild depression, slightly dull eyes, slow movements | Moderate depression, closed eyes, lethargic, reduced perception |
| Feeding Behavior | Normal feeding | Reduced feeding | Ceased feeding |
| Expectoration | None | None | White, thin sputum present |
| Rhinorrhea | None | None | Clear nasal discharge present |
| Fever | Normal body temperature | 39.5–40.5°C | 40.5–41.5°C |
| Dyspnea | Normal breathing | Mild tachypnea/slight abdominal/thoracic breathing: slightly increased respiratory depth | Marked abdominal/thoracic breathing: visible deepening and effortful breathing |
| Coughing | None | Infrequent (rare) | Moderate frequency (5-15 min intervals) |

**Table S2** Prevalence of PA(S184N) and PB2(E627K) among H3N2 influenza A viruses

| Virus Protein | Number | Percentage | Virus Protein | Number | Percentage |
| --- | --- | --- | --- | --- | --- |
| AIV-PB2(627E) | 437 | 99.54% | AIV-PA(184S) | 447 | 95.11% |
| AIV-PB2(627K) | 2 | 0.46% | AIV-PA(184N) | 4 | 0.85% |
| AIV-PB2(627 others) | 0 | 0% | AIV-PA(184 othSrs) | 19 | 4.04% |
| CIV-PB2(627E) | 318 | 97.85% | CIV-PA(184S) | 312 | 99.05% |
| CIV-PB2(627K) | 7 | 2.15% | CIV-PA(184N) | 3 | 0.95% |
| CIV-PB2(627 others) | 0 | 0% | CIV-PA(184 othSrs) | 0 | 0% |
| SIV-PB2(627E) | 3822 | 97.48% | SIV-PA(184S) | 3398 | 86.29% |
| SIV-PB2(627K) | 93 | 2.37% | SIV-PA(184N) | 102 | 2.59% |
| SIV-PB2(627 others) | 6 | 0.15% | SIV-PA(184 othSrs) | 438 | 11.12% |
| HuIAV-PB2(627E) | 528 | 0.51% | HuIAV-PA(184S) | 377 | 0.36% |
| HuIAV-PB2(627K) | 102115 | 99.44% | HuIAV-PA(184N) | 105308 | 99.63% |
| HuIAV-PB2(627 others) | 42 | 0.04% | HuIAV-PA(184 othSrs) | 12 | 0.01% |

**Table S3** Quality control assessment of single-cell RNA sequencing

| Sample | Control | GD14-ma[PA(S184N)+PB2(E627K)] | GD14-WT |
| --- | --- | --- | --- |
| Number of Reads | 354278336 | 339106691 | 360346835 |
| Valid Barcodes | 89.02% | 88.72% | 88.87% |
| Q30 Bases in CB+UMI | 96.89% | 96.81% | 96.01% |
| Q30 Bases in RNA read | 93.59% | 93.25% | 92.36% |
| Estimated Number of Cells | 5222 | 12152 | 8760 |
| Sequencing Saturation | 80.82% | 47.18% | 71.69% |
| Fraction Reads in Cells | 78.72% | 79.92% | 83.23% |
| Mean Reads per Cell | 67843.42 | 27905.42 | 41135.48 |
| Median Genes per Cell | 2063 | 2268 | 1790 |
| Total Genes Detected | 21728 | 23019 | 22004 |
| Median UMI Counts per Cell | 5101 | 5471.5 | 4067.5 |
| Confidently Mapping Reads per Cell | 38667.54 | 15369.51 | 24433.2 |
| Reads Mapped to Genome | 97.49% | 97.15% | 97.29% |
| Reads Mapped Confidently to Genome | 88.68% | 88.56% | 88.23% |
| Reads Mapped Confidently to Intergenic Regions | 5.37% | 3.85% | 4.35% |
| Reads Mapped Confidently to Intronic Regions | 13.75% | 12.15% | 11.51% |
| Reads Mapped Confidently to Exonic Regions | 69.55% | 72.55% | 72.37% |
| Reads Mapped Confidently to Transcriptome | 81.33% | 77.67% | 80.30% |
| Chemistry | BDHT | BDHT | BDHT |
| Include introns | TRUE | TRUE | TRUE |
| Transcriptome | mm10_Ensembl_Ensembl100 | mm10_Ensembl_Ensembl100 | mm10_Ensembl_Ensembl100 |
| Pipeline Version | STARSolo_2.7.10a | STARSolo_2.7.10a | STARSolo_2.7.10a |

**Table S4** Distribution and proportions of cellular subtypes across experimental groups

| Cluster | Control | Percentage | GD14-ma[PA(S184N)+PB2(E627K)] | Percentage | GD14-WT | Percentage | SUM |
| --- | --- | --- | --- | --- | --- | --- | --- |
| Epithelial | 134 | 2.98% | 62 | 0.66% | 18 | 0.24% | 214 |
| Endothelial | 717 | 15.97% | 1037 | 10.98% | 292 | 3.82% | 2046 |
| Fibroblast | 194 | 4.32% | 254 | 2.69% | 82 | 1.07% | 530 |
| SMC | 14 | 0.31% | 27 | 0.29% | 13 | 0.17% | 54 |
| Alveolar_Macrophage | 902 | 20.08% | 2735 | 28.95% | 1409 | 18.42% | 5046 |
| Neutrophil | 391 | 8.71% | 1239 | 13.11% | 2049 | 26.78% | 3679 |
| MoMac | 190 | 4.23% | 2098 | 22.21% | 597 | 7.80% | 2885 |
| DC | 90 | 2.00% | 309 | 3.27% | 183 | 2.39% | 582 |
| NK | 165 | 3.67% | 371 | 3.93% | 443 | 5.79% | 979 |
| B_cell | 494 | 11.00% | 224 | 2.37% | 431 | 5.63% | 1149 |
| T_cell | 1200 | 26.72% | 1092 | 11.56% | 2133 | 27.88% | 4425 |
| SUM | 4491 |  | 9448 |  | 7650 |  |  |
